# Amniotic Fluid Classification and Artificial Intelligence: Challenges and Opportunities

**DOI:** 10.3390/s22124570

**Published:** 2022-06-17

**Authors:** Irfan Ullah Khan, Nida Aslam, Fatima M. Anis, Samiha Mirza, Alanoud AlOwayed, Reef M. Aljuaid, Razan M. Bakr

**Affiliations:** Department of Computer Science, College of Computer Science and Information Technology, Imam Abdulrahman Bin Faisal University, P.O. Box 1982, Dammam 31441, Saudi Arabia; iurab@iau.edu.sa (I.U.K.); 2180007105@iau.edu.sa (F.M.A.); 2180007084@iau.edu.sa (S.M.); 2180004886@iau.edu.sa (A.A.); 2180004320@iau.edu.sa (R.M.A.); 2180005561@iau.edu.sa (R.M.B.)

**Keywords:** amniotic fluid (AF), artificial intelligence, deep learning, machine learning, oligohydramnios, polyhydramnios, ultrasound

## Abstract

A fetal ultrasound (US) is a technique to examine a baby’s maturity and development. US examinations have varying purposes throughout pregnancy. Consequently, in the second and third trimester, US tests are performed for the assessment of Amniotic Fluid Volume (AFV), a key indicator of fetal health. Disorders resulting from abnormal AFV levels, commonly referred to as oligohydramnios or polyhydramnios, may pose a serious threat to a mother’s or child’s health. This paper attempts to accumulate and compare the most recent advancements in Artificial Intelligence (AI)-based techniques for the diagnosis and classification of AFV levels. Additionally, we provide a thorough and highly inclusive breakdown of other relevant factors that may cause abnormal AFV levels, including, but not limited to, abnormalities in the placenta, kidneys, or central nervous system, as well as other contributors, such as preterm birth or twin-to-twin transfusion syndrome. Furthermore, we bring forth a concise overview of all the Machine Learning (ML) and Deep Learning (DL) techniques, along with the datasets supplied by various researchers. This study also provides a brief rundown of the challenges and opportunities encountered in this field, along with prospective research directions and promising angles to further explore.

## 1. Introduction

Amniotic fluid (AF) refers to a protective liquid surrounding the fetus inside the amniotic sac that plays essential roles in fetal development. Some of these roles entail shielding the baby from external pressure or sudden motion, ensuring optimal temperature, providing antibodies, and allowing the baby to begin exercising the muscles of various organ systems by floating inside the sac and swallowing the AF [1]. Normal volumes of AF, which typically lie in the range of 500–2000 mL, can cater to all the aforementioned functions [2]. However, abnormal volumes, typically lying outside the range of 500–2000 mL [2], can cause serious conditions, such as oligohydramnios, which refers to an insufficient amount of AF, and polyhydramnios, which is characterized by an excessive amount of AF. The occurrence of oligohydramnios leads to an increased risk of stillbirth or miscarriage [3]. Furthermore, it can sometimes lead to abnormalities, such as underdeveloped lungs, since the AF plays an essential role in lung development during the middle of the second trimester [3]; it can also lead to umbilical cord compression, contractures, etc. Similarly, the occurrence of polyhydramnios can also increase complications during pregnancy and delivery. In this condition, excessive amounts of AF can cause premature contractions, leading to early delivery, difficulty breathing, limited oxygen supply to the fetus due to the umbilical cord becoming trapped beneath the fetus, etc. [3]. Therefore, AF volume estimation is a fundamental measurement required to monitor fetal development.

A fetal ultrasound (US), or sonogram, is a technique used to examine the baby’s maturity and development, and is widely used to estimate AF volume by physicians. AF volume is usually measured by evaluating the four-quadrant AF Index (AFI) or the Single Deep Vertical Pocket (SDP) [4] technique. In order to determine AF levels, sonographers find a suitable AF pocket, and then trace the depth of the AF by locating an appropriate point. Despite the fact that AFI and SDP are known to be repeatable and semi-quantitative, manual AFI estimation is highly reliant on the sonographer’s skills and expertise [5]. Sometimes, despite having years of experience, sonographers find it difficult to accurately determine the AF volume in the fetus. Thus, complete assessment is a lengthy process, sometimes leading to inaccurate results. However, considering how technological advancements in the medical field have made a tremendous impact in treating many disorders that were once considered irreversible, we recognize that introducing a certain degree of automation in AF volume estimation could reduce errors and provide more reliable measurements. The automation process is where Artificial Intelligence (AI) comes into the picture.

AI techniques, namely Machine Learning (ML) and Deep Learning (DL), can be used to efficiently automate the process of AF volume estimation. ML and DL excel in visual pattern identification, making them particularly useful for sonographers [6]. Even though obstetric and gynecological ultrasonography are two of the most popular imaging procedures, ML and DL have made a limited impact in this field. However, they have a great deal of potential in aiding repetitive US operations, such as automatically selecting optimum images and providing instantaneous anatomical measurements. One of the most significant advantages to using these methods is that they aid the sonographer in analyzing the US images for AF volume estimation, and standardize the US technique to improve patient safety.

To the best of our knowledge, no paper in the literature so far has conducted a comprehensive review of the existing studies in this area of utilizing AI for AF detection and classification. We noticed that there was a need to conduct a comprehensive review of the existing studies so as to provide a starting point for researchers to identify the findings and gaps in this area and conduct more extensive research. Motivated by this need, in this paper we explore the existing literature to examine what is presently known about the numerous concepts and theories related to utilizing AI for AF identification from US images. This review paper makes the following key contributions:

- Summarizes the use of ML- and DL-based classification and segmentation methods in measuring and classifying AF volume.
- Discusses the factors leading to abnormal AF levels and summarizes the studies focusing on using AI to detect these factors.
- Summarizes the previous studies’ performances in terms of their detection approaches, including their accuracy, dice similarity, etc.
- Presents a comprehensive discussion on the techniques used by the previous studies and the dataset types and sizes.
- Highlights the challenges and future directions in this field of AF detection using AI techniques.

**Figure 1:**
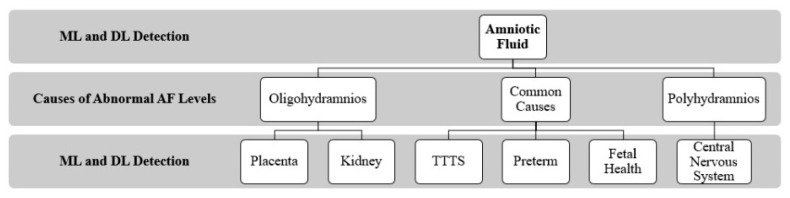
Taxonomy of AF detection using AI techniques.

Figure 1 presents the overall structure of the review paper, which includes discussing AF detection using ML and DL techniques, investigating the causes or factors leading to abnormal AF, and the ML and DL techniques adopted to detect these factors.

The paper is structured as follows: Section 2 presents the methodology of the paper. Section 3 investigates the AI-based techniques for diagnosis and classification of AF. Section 4 outlines the factors leading to abnormal AF levels, and reviews the studies conducted to investigate these factors using AI. Section 5 provides a discussion on the most widely used AI algorithms in the most-related papers, as well as the type and size of datasets analyzed in the literature. Section 6 presents the challenges and opportunities in this field. Finally, Section 7 provides a conclusion to summarize the results of our study.

## 2. Correlation between AF Levels and Gestational Age

During the first half of pregnancy, AF is derived from fetal and maternal compartments. With time, active secretion of fluids from amniotic tissues (epithelium) leads to the early formation of AF. By the second trimester, the fetus contributes to AF volume through urination. However, as the fetus ages, several factors, such as fetal swallowing, rigorous activity in the respiratory tract, continuous fluid exchange by hydrostatic or oncotic forces, etc., may contribute to AF elimination [7]. It is often argued that the meaning of the reduced fluid is different depending on gestational age. The amount of AF varies with gestational age, e.g., 50 mL at 12 weeks, 150 mL at 16 weeks, and then increases per week by 50 mL until 34 weeks [8]. Low AF levels are most prevalent during the final trimester. Overdue births are at higher risk of reduced AF levels as the AF level drops by 50% after 42 weeks.

A decrease in AF levels is usually observed at around a gestational age of 34 weeks [9]. A patient diagnosed with oligohydramnios may be at risk of many disorders and birth defects. In some cases, oligohydramnios could potentially cause child death. Patients showing signs of oligohydramnios in the first trimester are at an increased risk of abortion. In the second trimester, the degree of oligohydramnios is of prime significance during diagnosis; severe oligohydramnios could lead to fetal death, whereas borderline oligohydramnios is of lesser concern. In the third trimester, oligohydramnios is typically known to restrict fetal movement, therefore inducing umbilical cord compression and placing the fetus in a dangerous state.

## 3. Methodology

**Figure 2:**
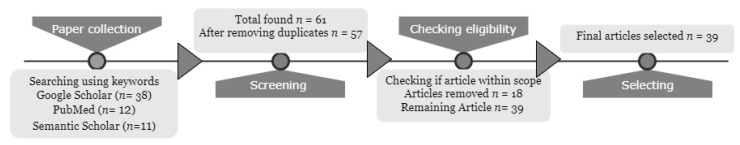
Methodology adopted for the systematic review.

The purpose of this review is to study the existing research in the domain of AF detection and classification using AI techniques. In order to formulate this review, the Preferred Reporting Items for Systematic Reviews and Meta-Analysis (PRISMA) method was adopted to search for and include the most-related studies, as illustrated in Figure 2.

As a first step, we searched for AF-related papers on various popular databases, namely Google Scholar, PubMed, and Semantic Scholar. To search for the papers, we used relevant keywords related to our scope, such as AF, twin-to-twin transfusion, fetal kidney/abdomen, placenta, DL, ML, AI, sonography, Image Analysis, etc., to name a few. Based on this step, 61 papers in total were found from the aforementioned databases. As a next step, we screened the collected papers, whereby we removed the duplicates. At the end of this step, we obtained 57 unique papers. Next, we checked the eligibility and the relevance of these papers to the scope of our review, and found that some did not fall within the scope. Finally, the number of most-relevant papers found was 39. All 39 papers were included in the review and discussed. The results and findings of the articles, and the future scope, are discussed in the present paper.

## 4. Artificial Intelligence-Based Techniques for Diagnosis and Classification of Amniotic Fluid

A number of studies have been published addressing the use of AI techniques in AF detection. This section reviews the existing literature on the topic by discussing various concepts and theories, including previous studies and their findings. The related works are organized based on the most relevant AF assessments with two main approaches: classification and segmentation.

### 4.1. Classification

Some studies concentrated on classifying AF from the fetal US images or videos for assessing fetal health. Following this ideology, Bahado-Singh et al. [10] compared the DL and ML models, namely Random Forest (RF) and the Support Vector Machine (SVM), and neural networks for AF detection by utilizing sonographic, clinical, and demographic factors to predict perinatal outcomes in pregnant women with a short Cervical Length (CL). The researchers used a combination of omics, demographic, and clinical data to study 26 samples. DL yielded a significant performance, with an Area Under the Curve (AUC) (0.85 Curie (CI)) of 0.890 (0.810–0.970) for delivery < 34 weeks of gestation, 0.890 (0.790–0.990) for delivery < 28 days post-amniocentesis, and 0.792 (0.689–0.894) for Neonatal Intensive Care Unit (NICU) admission. Additionally, R. Ramya and K. Srinivasan [11] introduced a hybridized strategy to create the Hybrid Bidirectional Unidirectional—Long Short-Term Memory (HBU-LSTM) algorithm by merging the unidirectional and bidirectional models. In US fetal images, different image preprocessing methods were used for distance prediction, i.e., Convolutional Neural Network (CNN) and bidirectional LSTM; the suggested model achieved the best results, with a Mean Squared Error (MSE) of 0.5244 and a Mean Squared Deviation (MSD) of 0.4554.

Following the same principles, Ayu et al. [12] used ML algorithms to classify AF into six categories: Oligohydramnion Clear, Oligodramnion Echogenic; Polygohydramnion Clear, Polygohydramnion Echogenic; Normal Clear and Normal Echogenic. The US images dataset was acquired from a local hospital, and it contained 95 US images. During preprocessing, cropping, and conversion of images from RGB to greyscale was applied to facilitate image segmentation. Additionally, the SDP feature was extracted. After the preprocessing steps, the images were classified based on rule-based SDP and the RF algorithm. The results obtained by the model had a good accuracy of 0.9052, outperforming many studies. In another study, Ayu and Hartati [13] investigated a pixel classification algorithm to differentiate AF regions on US pictures with noise, hazy edges, distortions, and poor contrast. The method involved local first-order statistical methods and data as gray-level to produce each pixel’s traits. Classification of each pixel was performed using RF and Decision Tree (DT) techniques to create four classes: AF, fetal body, placenta, and uterus. They found 5 × 7 window sizes for the RF method depicted the best performance, with 0.995 accuracy; these were obtained from a mode with 50 US testing images for the AF area’s segmentation.

Additionally, Amuthadevi and Subarnan [14] focused on measuring the AFI, as well as the geometry and shadowed properties of AF at various phases of gestation, and developed a fuzzy technique to help with forecasting anomalies in infant weight, head circumference (HC), and the requirement for critical care following delivery. The assessment of these factors would prove useful for delivery decisions, and ultimately aid in avoiding premature birth. US scans were used for this, and the condition of the patient was determined depending on the features obtained. Oligohydramnios, borderline, normal, or polyhydramnios were the classifications in the AFI. This classification helped practitioners gain insight into the pregnancy’s effect on both the child and the mother. The implemented fuzzy logic system was tested by comparing the results obtained algorithmically against a physician’s opinion on the AFI level, which was a 0.94 match, and the geometry of the AF images at various weeks of gestation; classifying them into one of the cases led to an accuracy of 0.925. Table 1 below presents a summary and comparison of the AF classification studies utilizing different AI techniques, which are subdivided into ML and DL.

### 4.2. Segmentation

Some studies focused on segmenting AF from US images by utilizing DL methods. For example, Cho et al. [5] established a DL-based model that detects AF pockets. The segmentation of an AF pocket with their proposed DL model, AF-net, is the most important phase. AF-net can be described as a version of U-net, along with a combination of three concepts: atrous convolution, multi-scale side-input layer, and side-output layer. The dataset used was comprised of 435 US images. Preprocessing methods included cropping, resizing images, and random contrast or brightness settings to lessen the influence of overfitting. To ensure effectiveness, five-fold cross-validation was applied. The proposed model accomplished a Dice Similarity Coefficient (DSC) of 0.877 ± 0.086 for AF segmentation, and a precision value of 0.898 ± 0.111. They found that a stable and consistent method is required to control varying transducer conditions. Moreover, there is a limitation in assessing AFIs in obese women due to the inflammation of adipose tissue.

Similarly, Sun et al. [15] aimed at estimating AF volume from US images. In order to segment the AF, they used DL models using a dual-path network. The dual net included the primary path that comprised of AF-net (similar to the previous study), and the secondary path that entailed an auxiliary network. The dataset includes 2380 US images acquired manually from a medical center. The dataset underwent preprocessing techniques, mainly cropping, resizing, normalization, and augmentation, as well as five-fold cross validation. The proposed network successfully achieved a DSC of 0.8599 ± 0.1074. Similarly, Li et al. [16] applied DL for the segmentation of the AF and tissue sections of fetal US images obtained from clinical examinations. The DL model was initiated by encoding the input image into down-scaled feature maps utilizing convolution and pooling stages. This was followed by decoding, achieved via the un-pooling layers in accordance with the 1 × 1 convolution kernels for acquiring the image of the input size. The dataset consisted of videos, 20 s in length, of four patients, from which the still images were sampled every two frames. By random separation, 900 training images and 400 testing images were attained. Different network settings were tested, confirming the three inner layers of the kernel achieved the best performance, with an accuracy of 0.93. However, more data and segmentation at the object border is required for the enhancement of the technique.

Additionally, Ayu et al. [17] proposed an AF segmentation model with 50 fetal B-Mode US images. The segmentation model applied pixel classification based on the RF method. For comparison, the images were taken at two window sizes (3 × 3 and 5 × 5), then a Radiologist Expert labeled multiple points according to 3 classes (AF, fetal body, uterus). As a result, images with a window size of 5 × 5 had an accuracy of 0.8586 and 0.8145 for images with window size 3 × 3 In another study, Ayu et al. [18] focused on AF segmentation using a pixel classification model which applied various classifiers namely RF, DT, Naive Bayes (NB), SVM, and K-Nearest Neighbor (KNN) to classify the AF from other objects in the images. The dataset was acquired from a local hospital and consisted of 55 US images. The sampling window technique was used to construct training sets, producing pixel information specific to certain regions. The results of the proposed model were best achieved using the RF classifier, which obtained a DSC of 0.876 and pixel accuracy of 0.857.

Furthermore, Looney et al. [19] proposed a multiclass CNN model to segment the placenta, AF, and fetus. The dataset consisted of 2093 labeled placental volumes augmented by 300 volumes with placenta, AF, and fetus annotated for multi-class segmentation. For the Placenta Segmentation (PS) model, out of 2093 images were used and the model used was Fully CNN (FCNN). For multiclass segmentation, a two-pathway Hybrid Model (HB) was built using the remaining 300 cases from the original dataset. For the PS model, the highest obtained results were obtained with a DSC of 0.85 after 17,000 training steps. In the case of the HB model, it improved the placental segmentation due to the dual pathway and exhibited a DSC of 0.84. Correspondingly, Anquez et al. [20] studied the Utero-Fetal Unit (UFU) segmentation of 19 3D US images in the first trimester of the fetal. The main goal was to extract fetal tissues and AF automatically. The study used exponential and normal distributions for saturated images, whereas Rayleigh and normal distributions were used for non-saturated images. In addition, the Gamma distribution was used as a generic formulation. The study has achieved an accuracy average of 0.89. Table 2 below presents summary and comparisons of the AF segmentation studies utilizing different AI techniques subdivided into ML and DL.

## 5. Factors/Causes of Abnormal Amniotic Fluid Levels

Oligohydramnios is an AF abnormality that causes a reduction in AF volume for the early stage of pregnancy. Inadequate AF levels can be caused by a variety of obstetric, fetal, or placental problems, all of which result in negative fetal consequences [21]. Polyhydramnios is a condition in which the AF level rises during gestation, a condition that is linked to higher maternal and neonatal mortality rates [21]. In this section, we review the studies that focus on using ML/DL to detect the factors or elements in the fetus that possibly contribute to causing abnormal AF levels. The breakdown of this section is already presented in Figure 1.

### 5.1. Oligohydramnios

Through extensive research, it appears that oligohydramnios is mostly caused by placental abnormalities, followed by congenital anomalies. The following section summarizes the most prevalent studies regarding the factors affecting oligohydramnios.

#### 5.1.1. Placenta

Considering the idea that the placenta is essential in determining the fetal heath, as well as useful in determining its contribution to high AF levels, some studies focused on segmenting or detecting placenta in fetal images. Following this idea, Han et al. [22] proposed an automatic segmentation method using U-net, a CNN for biomedical image data. The dataset, acquired from a local hospital, contained 1110 Magnetic Resonance Imaging (MRI) images, which underwent a preprocessing technique mainly featuring normalization. The overall accuracy achieved by the model was 0.98. In another study, Yang et al. [23] utilized 3D US images for automatic semantic segmentation of the placenta, as well as gestational sac and fetus. Based on 3D FCNN, they proposed a Recurrent Neural Network (RNN) to flexibly explore 3D semantic knowledge from a sequential perspective. The dataset used contained volumetric US images from 104 pregnant women, and it underwent augmentation. The FCNN achieved a good DSC of 0.882. Furthermore, Hu et al. [24] utilized a CNN to segment placenta from US images using a dataset that contained 1364 fetal images. In order to identify artifacts specific to US, the CNN contained a novel layer weighted by automated acoustic shadow detection. The CNN built with the additional layer achieved a DSC of 0.92. Similarly, Zimmer et al. [25] followed a multi-task approach, and used transfer learning to identify placenta position in U-net architecture. The dataset used contained real-time 1054 3D US images, and it underwent augmentation. The achieved segmentation accuracy was improved, with the highest DSC of the anterior and posterior being 0.87 and 0.81, respectively.

However, sometimes placental-mediated diseases are not recognized until later stages. Hence, Hu et al. [26] proposed a CNN pipeline to detect the presence of placental diseases. The model consists of segmentation of the placenta followed by classification utilizing a dataset containing US images of 321 patients (13,384 frames). The model successfully achieved a high classification accuracy of 0.81. In another study, Schilpzand et al. [27] focused on detecting low-lying placenta or placenta previa in resource-limited settings using US images. The authors segmented the placenta using U-net, achieving a DSC of 0.84, utilizing 6576 US images. Then, classification was applied to differentiate it as normal or placenta previa, which achieved a specificity of 0.82 in 148 cases. Additionally, Zimmer et al. [28] proposed a method using 3D US images to segment the placenta at late gestation using 3D CNN. The dataset contained about 127 3D US images, and the model successfully performed multi-view PS, achieving a DSC of 0.8. Additionally, Looney et al. [29] examined a Deep CNN (DCNN), known as DeepMdic, constituting of a ground truth based on a semi-automated random walker method output. For segmentation of the 3D US data, the input volume was converted into a subjective, undirected graph, and a segmentation protocol was then applied. The dataset consisted of placenta from 3064 cases during the first trimester. The 300 test cases produced a DSC of 0.73. In another study, Looney et al. [30] proposed a unique technique to atomize placental segmentation from 3D US volumes controlled by a complete CNN, OxNNet, for ground truth. Adverse pregnancy outcome, namely Small for Gestational Age (SGA) prediction was evaluated using a CNN containing data for 3104 cases. The results obtained showed the DSC to vary from 0.73 to 0.81.

Furthermore, Saavedra et al. [31] utilized portable technology to propose a model for the automated evaluation of placenta previa throughout the last trimester in remote regions. The method includes PS by U-net DL algorithm applied to 11,014 US images from 10 patients, and identifies the placenta position. The approach was found to have a sensitivity of 0.75 and a specificity of 0.92. Similarly, Oguz et al. [32] presented a new technique for automatic PS in 3D US images that combines Joint Label Fusion (JLF)-based multi-atlas segmentation and DL approaches. The procedure was tested on a sample of 47 patients during the first trimester by merging the JLF and CNN models. With 86.3 ± 5.3 as the mean dice coefficient, the model indicates a significant improvement. Moreover, for recognizing uteroplacental interface in US images, Qi et al. [33] introduced Knowledge-guided Pretext Learning (KPL), which trains anatomical structures without employing external data, much like ImageNet. The dataset consisted of 101 placental 3D US volumes. KPL had the greatest performance of Optimal Dataset Scale (ODS), achieving 0.605 with VGG-19, while ImageNet had good results as well. Correspondingly, Romeo et al. [34] suggested that ML assessment utilizing MRI-based texture features would be a useful method for detecting placental tissue anomalies that underpin the placenta accreta spectrum in women with placenta previa. MRI tests of 64 individuals were used to test the various algorithms, with KNN achieving the greatest accuracy of 0.981. Likewise, to address the challenges of automated placental structural classification, Chen et al. [35] introduced a new transfer learning model, PlacentaNet, comprised of multiple encoder–decoder CNNs. With a dataset covering photographic images of 1003 placentas, the proposed model achieved a total classification accuracy of 0.9751.

#### 5.1.2. Kidneys

The occurrence of oligohydramnios during pregnancy has been identified as a significant risk factor for renal damage. Thus, Sridar et al. [36] began with prenatal US images and identified 14 angles using the AlexNet neural network, which had been trained with a genuine visual dataset. They devised a method for training two adjacent networks at the same time. One network’s feed was the full US image, which was utilized to understand the image’s content overall. The second network’s feed consisted of random confined segments of US images, which was necessary to identify the image’s specific and distinct characteristics. They had a 0.97 mean accuracy, 0.7647 precision, and 0.7541 mean recall rate.

### 5.2. Polyhydramnios

Although polyhydramnios may be classified as a rare disorder [21], it most definitely can lead to dire consequences, such as child mortality. This section summarizes the literature on some of the factors resulting from this disorder.

#### Central Nervous System

The most prevalent anomalies linked to polyhydramnios are those of the central nervous system. Therefore, Zhou et al. [37] applied DL neural network algorithm to diagnose fetal central nervous system malformation. The proposed algorithm aims to optimize analysis and identify malformation from a set of 63 US images from pregnant women. The proposed model utilizes the CNN method and was able to improve the accuracy of the results; nevertheless, due to the small size of the dataset, the results could still be improved.

Similarly, Attallah et al. [38] developed a new approach for detecting fetal neurological abnormalities using DL methods. Transfer learning, deep feature extraction, feature reduction, and classification are the phases of the methodology. There are 227 embryonic MRI scans in the collection (113 are normal and 114 have neurological abnormalities), with gestational age spanning from 16 to 39 weeks. Using quadratic SVM classifiers trained with deep features taken from AlexNet and ResNet50 combined, the accuracy was 0.886. The findings demonstrate that the presented approach may successfully detect fetal neurological abnormalities from prenatal MRI data at diverse stages of pregnancy.

### 5.3. Common Factors of Oligohydramnios and Polyhydramnios

The following subsections demonstrate common factors that may contribute to both oligohydramnios and polyhydramnios.

#### 5.3.1. TTTS (Twin-to-Twin Transfusion Syndrome)

TTTS occurs when one of a set of monochorionic diamniotic twins suffers severe polyhydramnios during the middle of gestation, which results in the co-twin developing oligohydramnios. For this reason, Bano et al. [39] proposed a combined CNN and LSTM model to study fetoscopic videos captured from different human TTTS cases; the dataset consists of 138,780 frames. The proposed model achieved a precision value of 0.96, and the results show that the proposed model handled the challenges in the fetoscopic environment better than other methods and resulted in improved prediction for multi-label frames. Moreover, Bano et al. [40] also presented a DL-based mosaicking framework for fetoscopic videos captured from different environments. In total, 2400 frames were extracted to capture different environments, including simulation, phantom, ex vivo, and in vivo environments. A comparison was performed with existing feature-based and deep image homography methods, and the proposed model demonstrated its robustness and outperformed the existing methods.

Correspondingly, Ahmad et al. [41] introduced a new shared control approach for fetoscopic applications. The model trained 30,000 images using CNN to predict the relative orientation of the placental surface made by a single monocular fetoscope camera photo, and achieved an accuracy of 0.87 for the simulated dataset. Finally, Casella et al. [42] aimed to use DL in providing automatic and fast membrane segmentation in fetal images. They used an adversarial network consisting of two CNNs. The dataset included 900 images acquired and labeled from six surgical videos (150 frames per video). After training and validating, the adversarial network obtained a DSC of 0.9191.

#### 5.3.2. Preterm

One of the pregnancy complications that motivates researchers to explore ML solutions is Prelabor Rupture of Membranes (PROM). Sufriyana et al. [43] aimed to predict the likelihood of PROM and delivery time. There were five ML and statistical techniques compared: Ridge Regression (RR), Elastic Net Regression (ENR), RF, Gradient Boosting (GB) models, and the Deep-Insight Visible Neural Network (DI-VNN). The study showed that RF has the capability to estimate delivery time, unlike the other models. However, the DI-VNN classifier showed outstanding performance, with a sensitivity of 0.494 and specificity of 0.816. Hence, it was chosen to be integrated as a web application. Extreme Preterm Birth (EPB) is another condition that causes early delivery before the 28th week. Gao et al. [44] focused on developing a DL model for EPB using RNN, comparing the performance with Linear Regression (LR), SVM, and GB models. The number of patients who participated was 25,689, containing approximately 0.0 EPB cases. The model resulted in an AUC of 0.827 and a sensitivity of 0.965.

#### 5.3.3. Fetal Health

Along with gestational age, fetal biometry and weight estimation are indicators of babies’ and their mothers’ short- and long-term health. Several studies have been published to automate the identification of any potential risks. Lee et al. [45] have experimented with multiple ML approaches to predict a newborn’s Body Mass Index (BMI) using US images. The study contained 3159 patients across several countries, with a range of gestational age from week 21–36 and later. The dataset has been tested using LR, RF, and an Artificial Neural Network (ANN) with one, two, and three hidden layers, resulting in RF having the highest average of MSE of 2.1610. In addition, they found that Abdominal Circumference (AC) and Estimated Fetal Weight (EFW) in week 36 or later are primary predictors of the newborn’s BMI. Similarly, Feng et al. [46] presented a model for EFW based on SVM and Deep Belief Network (DBN) models. The number of pregnant women who have participated in the study is 7875, including High Birth Weight (HBW) and Low Birth Weight (LBW) fetuses. The results show Mean Absolute Percent Error (MAPE) ranges of 0.0609 ± 0.0506 and a Mean Absolute Error (MAE) of 98.55 ± 158.63 g. Correspondingly, Oghli et al. [47] present CNN architecture based on Multi-Feature Pyramid U-net (MFP-U-net) to measure fetal biometry, including Biparietal Diameter (BPD), HC, AC, and Femur Length (FL) using 1334 US images. The model achieved 0.98 on DSC, 1.14 mm on Hausdorff Distance (HD), 0.95 on conformity, and 0.2 mm on Average Perpendicular Distance (APD). Table 3 summarizes AI studies focusing on investigating the causes of factors of abnormal AF Levels.

## 6. Discussion

**Figure 3:**
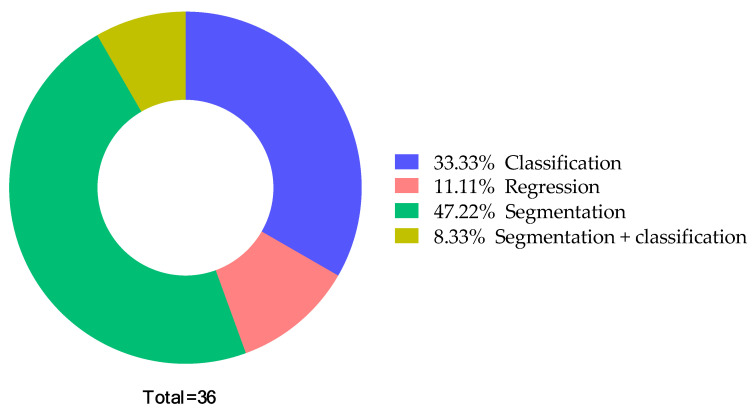
Distribution of the studies based on the AI method type.

**Figure 4:**
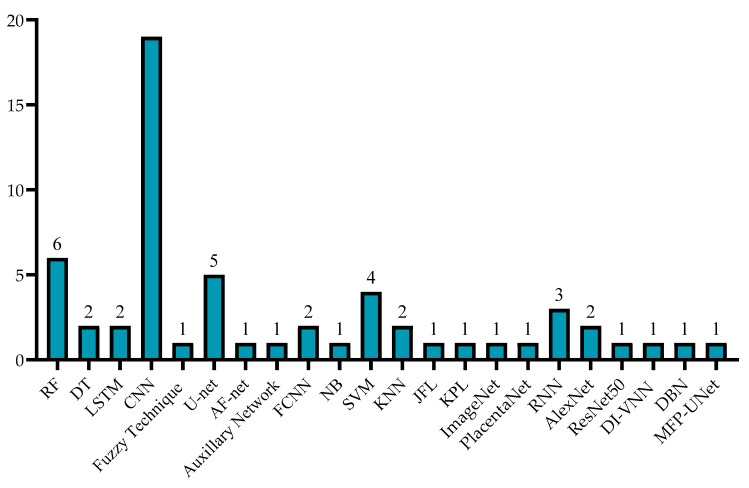
AI techniques used.

**Figure 5:**
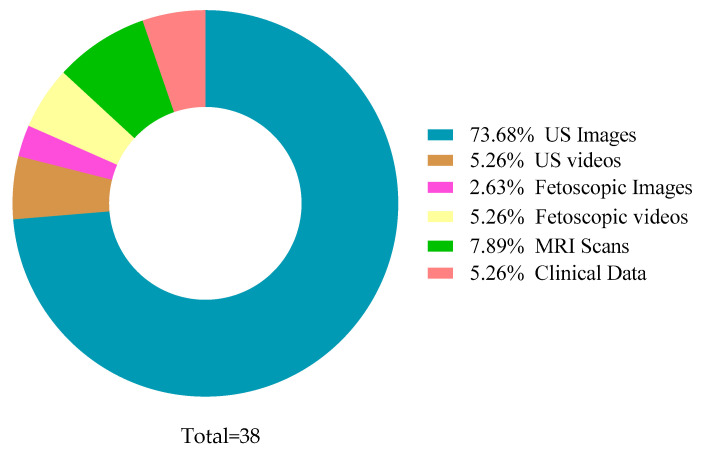
Data types used by various studies.

A number of studies have been published addressing the use of DL techniques for fetal health assessment that utilized segmentation and classification. Almost all the researchers assessed utilizing US images as inputs for their models. Since some studies suffered from small dataset sizes, they used the augmentation technique. Figure 3 shows the type of method used for Amniotic Fluid. While Figure 4 illustrates the type of techniques used like list of ML, DL techniques used by the previous studies, and Figure 5 illustrates the datatype used for diagnosis adopted by the studies. As shown in Figure 3, the most widely used method among the various studies was segmentation, and the least was regression Furthermore, as seen in Figure 5, RF and CNN were widely used methods by a plethora of authors and they tend to exhibit a good performance among the literature investigated in this report. U-net is also a form of CNN architecture for biomedical imaging that provides fast and accurate segmentation of images.

**Figure 6:**
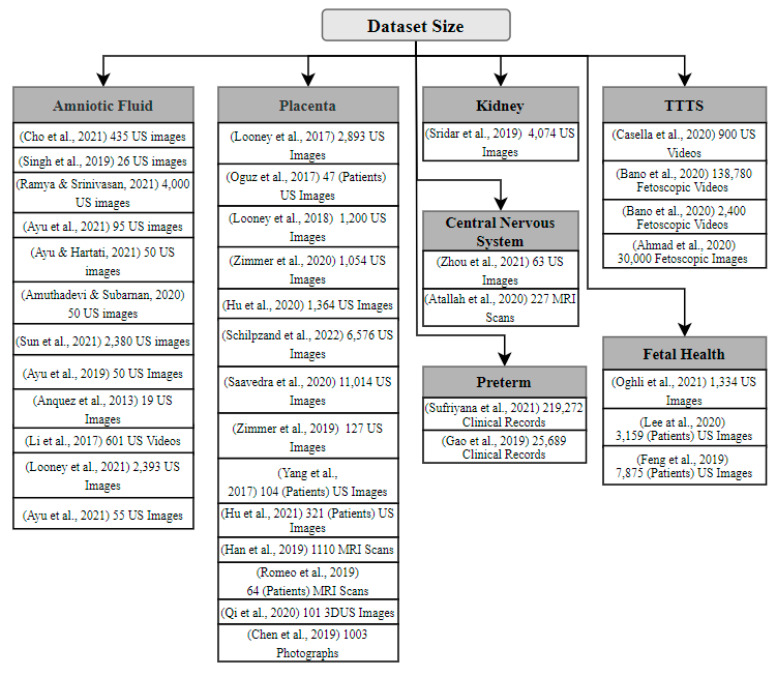
Dataset type and size of various studies.

Most of the reviewed papers have used fetal 2D and 3D US images and videos, MRI images, fetoscopic images and videos, and clinical data as their datasets. The primary focus was AF detection; nevertheless, fetal health factors were considered to understand the relation in-depth, including placenta, kidneys, abdomen, central nervous system, TTTS, preterm. Additionally, the datasets varied from 19 to 219,272 instances, with an average of approximately 12,392. Some datasets have used 3 × 3 to 5 × 5 pixels with 1 × 1 convolution in terms of the image window size. Figure 4 and Figure 6 summarize the reviewed dataset sizes and the types.

## 7. Challenges and Opportunities

Implementing AI techniques in fetal health has been proven to exhibit significant results for diagnosis. However, some aspects of this implementation are quite complex, and require further intervention to be applied safely and effectively in clinical applications. In this section, we highlight the general challenges and opportunities associated with the current research, and Table 4 provides a summarized view of the limitations and solutions in the reviewed literature.

### 7.1. Limited Dataset Size

Open-source datasets in the field of fetal medical imaging are scarce and, therefore, in most cases, researchers must obtain their own datasets through the assistance of a cooperating hospital or medical facility. The privately acquired dataset is often relatively small and, therefore, may contribute to lower accuracy in detecting fetal structures or characteristics. Consequently, datasets may be imbalanced, resulting in great differences between the distribution of classes. Training an algorithm on an unbalanced dataset often leads to a biased result.

Transfer learning is one of the most often-employed strategies used by researchers nowadays to overcome the issue of limited-sample datasets [48]. In addition, the availability of an open-source US image dataset would allow researchers to compare the performance of their proposed classification algorithms in a more efficient manner.

### 7.2. Sonographer Dependency

A sonographer’s subjectivity contributes to inconsistency in the collection of US images. This may worsen the restricted generalization ability of present DL-based algorithms. The practitioner’s method of obtaining US images affects the actual results [49]. As a result, AI algorithmic outcomes are determined by how the practitioner identifies the target object in the image, as well as whether the target object is successfully characterized and recorded. Hence, even for highly intelligent AI systems to perform effectively, the medical practitioner must have some level of expertise, at the very least sufficient to examine the patient appropriately.

To reduce the impact an individual sonographer has, sonographers may be further categorized according to their expertise and skill level. This ensures that novice sonographers may not capture an US image without the guidance of an expert sonographer to correctly identify target fetal planes.

### 7.3. Overfitting

The low universal applicability of AI algorithms for medical diagnosis and prediction (i.e., large variations in AI accuracy between patients) is a notorious problem, which is sometimes referred to as “overfitting” [49]. As US examinations are frequently performed in a variety of medical settings and on a wide range of patients, and are conducted by a broad set of clinical personnel with varying skill levels, overfitting may pose a severe danger to the accuracy of AI algorithms. In addition, ultrasonography equipment is highly varied compared to Computerized Tomography (CT) scans or MRI systems, with more suppliers and versions.

Solving the problem of overfitting emphasizes the significance of a thorough external validation of an AI system in the many clinical situations where it is expected to be applied [49]. Sonographers or medical professionals are encouraged to compare AI system findings with their own findings to ensure the accuracy of the AI algorithm, and thus to guarantee that it can be applied safely and in an effective manner.

### 7.4. Black Box Nature

Obstetrics and gynecology is a field that highly depends on the data generated from radiology, ultrasound, and CTG scans. The radiologically generated data is image-based and usually requires a highly sophisticated algorithm for automated diagnosis. In ML, there is usually a tradeoff among the predictive performance and the interpretability of the algorithm. Highly interpretable models, such as DT, LR, and NB, do not have the capability to understand the complex problems. On the other hand, the ensemble and DL models produce significant results in terms of prediction, but have a black box nature.

Due to the black box nature, clinicians are usually reluctant to adopt AI-based solutions in real life [50]. Therefore, clinicians tend to trust their perception more than outcomes generated by black box algorithms. Explainable AI has introduced transparency into the decision-making process and incorporates trust; clinicians can determine how the decision has been made.

### 7.5. Bias

AI-based models highly rely on the dataset that was used to train the model. This leads to the risk of bias. For example, if the model was trained using the dataset from one health center, then it will incorporate the bias from the data generated in that center. Thus, the model will not be generalized. Most of the previously defined studies used datasets from a single center. For the AI-based prediction model to be more generalized and robust, the model must be trained using multi-center and heterogenous data.

### 7.6. Distributed Data

One of the challenges AI systems have faced is decentralized data. Sometimes the diagnosis has been made to consider the data from different sources, such as radiology, pathology, and through physical examinations. Furthermore, the patients generally visit more than one hospital or health center, which leads to the division of data among multiple health centers. Similarly different health centers save the data in different formats. The decentralization of data raises the chance of errors, and data inconsistency and incompleteness.

### 7.7. Privacy and Cyber Security Concerns

AI-based diagnosis and prognosis systems require large amounts of data to better train the models. One of the major concerns is the patient’s privacy. Some patients refuse to provide data due to fear of privacy. Furthermore, the rise in automated diagnosis and health monitoring using the internet of things (IOT) increases the risk of cyber security threats. 

## 8. Future Research

With respect to all the reviewed literature in this broad field, we propose that further research should bridge many of the gaps that are currently encountered:

### 8.1. Image Quality

One of the weakest links in the deployment of AI in fetal health assessment is the poor image quality resulting from either inexperienced medical practitioners or an unsuitable fetal position within the placenta. There are areas of great potential improvement when enhancing the quality of captured US images.

### 8.2. Imaging Techniques

AI can be utilized to aid various advanced US methods, such as color Doppler imaging [7]. Gaining more views or angles to analyze an image would be promising in terms of reaching an accurate interpretation of the image.

### 8.3. Awareness and Training

Acquainting and educating sonographers with the AI tools and/or methods used to automatically estimate fetal health indicators, such as AFV, ultimately escalates the performance of these AI-based solutions because sonographers will gain a better understanding of the consequences that a poorly captured US image can cause, especially in terms of patient misdiagnosis. In turn, sonographers will properly consider the aspects of each image taken, and whether the image meets the criteria of the algorithm for accurate assessment.

## 9. Conclusions

This paper attempted to provide a comprehensive review of the existing studies in the area of utilizing AI for AF detection and classification to provide a starting point for researchers to identify the knowledge gaps in this area and conduct more extensive research. Furthermore, it summarizes the use of DL- and ML-based classification and segmentation methods in measuring AF volume and highlights the challenges and opportunities in this field. Additionally, it discusses the various factors and causes leading to abnormal AF levels and reviews the studies focusing on using AI techniques to investigate these factors. Several ML and DL methods were analyzed for AF detection, including CNN, AF-net, RF, DT, NB, SVM, KNN, LSTM, etc. We created visual aids to analyze the reviewed papers based on the nature of their datasets as well as the performance of applied algorithms. From the analysis, we discovered that the most frequently used techniques in AF detection are the RF and CNN, which exhibited the highest performance among the literature investigated in this article. Furthermore, we discussed and listed some of the challenges, as well as recommended solutions to be utilized by interested parties for developing and funding the medical technology sector. Finally, we also discussed the future directions to undertake in order to advance this field of using AI techniques in the medical analysis of fetal health.

## Figures and Tables

**Table 1:** Summary of amniotic fluid classification studies using AI techniques.

Domain	Ref.	Year	Data Type	Dataset Size	Binary/ Multi-Class	Augmentation	Methods	MSE	Accuracy
ML	[12]	2021	US Images	95	Multi	No	RF		0.9052
	[13]	2021	US Images	50	Multi	No	RF, DT		0.995
DL	[11]	2021	US Images	4000	Multi	No	HBU-LSTM	0.5244	
	[10]	2018	US Images	26	Binary	Yes	CNN		0.95
	[14]	2020	US Images	50	Multi	No	Fuzzy Technique		0.925

**Table 2:** Summary of AF segmentation studies using AI techniques.

Domain	Ref.	Year	Data Type	Dataset Size	Binary/ Multi-Class	Augmentation	Methods	DSC	Accuracy
DL	[5]	2021	US Images	435	Binary	Yes	U-net (CNN)	0.877	
	[15]	2021	US Images	2380	Multi	Yes	AF-net + Auxiliary Network	0.8599	
	[19]	2021	US Images	2393	Binary, Multi	No	FCNN	0.84	
	[16]	2017	US Videos	601	Multi	Yes	CNN, Encoder–Decoder Network		0.93
ML	[18]	2021	US Images	55	Binary	No	RF, DT, NB, SVM, and KNN	RF—0.876	
	[17]	2019	US Images	50	Multi	Yes	RF	0.5553	0.8586
	[20]	2013	US Images	19	Binary	No	Bayesian Formulation	Overlap Measure—0.89	

**Table 3:** Summary of studies investigating abnormal amniotic fluid causes using AI techniques.

Condition	Ref.	Type	Year	Data Type	Dataset Size	Domain	Methods	Performance Measure
Oligo	[29]	Placenta	2017	US Images	2893	DL	CNN, Random Walker	DSC-0.73
	[22]	Placenta	2019	MRI Images	1110	DL	CNN (U-net)	Acc-0.98
	[30]	Placenta	2018	US Images	1200	DL	CNN	DSC-0.81
	[25]	Placenta	2020	US Images (3D)	1054	DL	U-net	DSC-0.87
	[24]	Placenta	2019	US Images	1364	DL	CNN	DSC-0.92
	[26]	Placenta	2021	US Images	321 (patients)	DL	CNN	Acc-0.81
	[27]	Placenta	2021	US Images	6576	DL	U-net	Acc-0.84
	[31]	Placenta	2020	US Images	11,014	DL	U-net	Sen-0.75 Spe-0.92
	[32]	Placenta	2018	US Images	47 (patients)	DL	JLF + CNN	DSC-0.863
	[33]	Placenta	2020	US Volumes	101	DL	KPL, ImageNet	ODS-0.605
	[34]	Placenta	2019	MRI Images	64 (patients)	ML	KNN	Acc-0.981
	[35]	Placenta	2019	Photographic Images	1003	DL	CNN (PlacentaNet)	Acc-0.9751
	[23]	Placenta	2017	US Images (3D)	104 (patients)	DL	FCNN, RNN	DSC-0.882
	[28]	Placenta	2019	US Images (3D)	127	DL	CNN	DSC-0.8
	[36]	Kidney	2019	US Images	4074	DL	CNN (AlexNet)	Acc-0.9705
Poly	[38]	Neuro	2020	MRI Images	227	DL	SVM + CNN (AlexNet, ResNet50)	Acc-0.886
	[37]	Neuro	2021	US Images	63	DL	CNN	*p* < 0.05
Both	[42]	TTTS	2019	US Videos	900	DL	CNN	DSC-0.9191
	[39]	TTTS	2020	Fetoscopic Videos	138,780	DL	CNN and LSTM	Precision-0.96
	[40]	TTTS	2020	Fetoscopic Videos	2400	DL	CNN	-
	[41]	TTTS	2020	Fetoscopic Images	30,000	DL	CNN	Acc-0.87
	[43]	Preterm	2021	Clinical Data	219,272 (patients)	DL	DI-VNN	Sen-0.494 Spe-0.816
	[44]	Preterm	2019	Clinical Data	25,689 (patients)	DL	RNN	Sen-0.819 AUC-0.777
	[45]	Health	2021	US Images	3159 (patients)	ML	RF	MSE-0.02161
	[46]	Health	2019	US Images	7875 (patients)	ML	SVM, DBN	MAPE-0.0609
	[47]	Health	2021	US Images	1334	DL	CNN (MFP-U-net)	DSC-0.98

**Table 4:** Limitations and solutions of amniotic fluid studies.

Ref.	Limitations	Solutions
[18]	Limited features to differentiate between actual AF and reflected waves. Insufficient uneven window shapes for relevant results.	Considering AF coordinates. Adding data for uneven and rectangular window shapes.
[12]	As there is no accurate measuring number for echogenicity, only doctor’s insight applied in observing the gray texture in US images.	-
[11]	Fetal weight ignored for results.	Present findings considering fetal weight.
[20]	Not entirely automated—Images manually labeled. Advance dataset with all different forms of US images is required.	Automize labelling of US images.
[13]	Uncertain aspects, such as angle and direction of the transducer, are ignored. Decreased AF appearance in overweight women due to US beam.	Considering maternal position during Amniotic Fluid Volume (AFV) measurement.
[5]	Errors in final finding due to secondary path in complementation procedure. US Images with noise disturbance.	Distinction between both paths. Unified image settings.
[15]	Moderate accuracy. Specific situation.	More broad case with additional clinical data and adequate labels.
[16]	Average accuracy.	-
[17]	Clinical data and US images exhibit moderate accuracy.	Omics data analysis to provide understanding to patients and help with clinical management.
[10]	Various measurements scattered.	First trimester screening tool combining different measures and characteristics.
[19]	Manual refinement of models.	Automate procedure.

## Data Availability

Not applicable.
